# Geographical, temporal and racial disparities in late-stage prostate cancer incidence across Florida: A multiscale joinpoint regression analysis

**DOI:** 10.1186/1476-072X-10-63

**Published:** 2011-12-05

**Authors:** Pierre Goovaerts, Hong Xiao

**Affiliations:** 1 BioMedware, Inc., Ann Arbor, MI, USA; 2 College of Pharmacy and Pharmaceutical Sciences, Florida A&M University, Tallahassee, FL, USA

## Abstract

**Background:**

Although prostate cancer-related incidence and mortality have declined recently, striking racial/ethnic differences persist in the United States. Visualizing and modelling temporal trends of prostate cancer late-stage incidence, and how they vary according to geographic locations and race, should help explaining such disparities. Joinpoint regression is increasingly used to identify the timing and extent of changes in time series of health outcomes. Yet, most analyses of temporal trends are aspatial and conducted at the national level or for a single cancer registry.

**Methods:**

Time series (1981-2007) of annual proportions of prostate cancer late-stage cases were analyzed for non-Hispanic Whites and non-Hispanic Blacks in each county of Florida. Noise in the data was first filtered by binomial kriging and results were modelled using joinpoint regression. A similar analysis was also conducted at the state level and for groups of metropolitan and non-metropolitan counties. Significant racial differences were detected using tests of parallelism and coincidence of time trends. A new disparity statistic was introduced to measure spatial and temporal changes in the frequency of racial disparities.

**Results:**

State-level percentage of late-stage diagnosis decreased 50% since 1981; a decline that accelerated in the 90's when Prostate Specific Antigen (PSA) screening was introduced. Analysis at the metropolitan and non-metropolitan levels revealed that the frequency of late-stage diagnosis increased recently in urban areas, and this trend was significant for white males. The annual rate of decrease in late-stage diagnosis and the onset years for significant declines varied greatly among counties and racial groups. Most counties with non-significant average annual percent change (AAPC) were located in the Florida Panhandle for white males, whereas they clustered in South-eastern Florida for black males. The new disparity statistic indicated that the spatial extent of racial disparities reached a peak in 1990 because of an early decline in frequency of late-stage diagnosis observed for black males.

**Conclusions:**

Analyzing temporal trends in cancer incidence and mortality rates outside a spatial framework is unsatisfactory, since it leads one to overlook significant geographical variation which can potentially generate new insights about the impact of various interventions. Differences observed among nested geographies in Florida show how the modifiable areal unit problem (MAUP) also impacts the analysis of temporal changes.

## Background

Inequity in overall cancer survival by race is recognized and attributed to differences in the stage at which cancer is diagnosed, its treatment, and, to a lesser extent, in the aggressiveness of tumors. Given equal treatment, there is considerable evidence that African Americans and Whites could experience equal stage-specific survival [1-3]. Still, Whites are diagnosed at earlier stages than African Americans for thirty-one of the thirty-four tumor sites [4]. In particular, although the racial differences in participation in prostate cancer early detection programs are narrowing [5] elderly Blacks are substantially less likely to undergo PSA screening than elderly Whites, a difference that is not completely explained by differences in socioeconomic status and comorbid conditions [6]. Although cancer specialists remain deeply divided over the effectiveness of the PSA blood tests as a diagnostic tool for prostate cancer, some mathematical models projected that 45% to 70% of the observed decline in prostate cancer mortality could be plausibly attributed to the stage shift induced by PSA screening [7]. Other studies found however that racial disparity in PSA testing is probably not a major factor behind current racial differences in prostate cancer mortality rates and declines [8]. As stressed by Nancy Krieger [9] in her paper on social disparities in cancer, "*research is needed to improve monitoring of these disparities. Data on population trends not only reveal whether health inequalities are increasing or decreasing over time, but also stands as critical tests of our etiologic hypotheses. More specifically, if we cannot explain the observed patterns, our knowledge is likely incomplete and our interventions potentially misguided*".

Joinpoint regression [10], also known as piecewise linear regression, is increasingly used to identify the timing and extent of changes in time series of health outcomes, thanks to a public-domain software developed at the US National Cancer Institute, NCI (http://srab.cancer.gov/joinpoint/). The basic idea is to model the time series using a few continuous linear segments. These segments are joined at points called joinpoints which represent the timing (i.e. year) for a statistically significant change in rate trend. The number of joinpoints, as well as the parameters of the piecewise linear regression, are estimated through an iterative procedure that tests whether models of increasing complexity (i.e. including more joinpoints) provide a significantly better goodness-of-fit than simpler models. The tests of significance use a Monte Carlo Permutation method. The approach yields estimates of average annual percent change (AAPC) which allows the summary and comparison of trends over a specified time interval [11]. Two segmented linear regressions (e.g. time trends for two ethnic groups) can also be compared and the parallelisms or identity of the two regression models can be tested [12].

There have been a few applications of joinpoint regression in cancer research, and it is now increasingly used to characterize long-terms trends in cancer mortality in the US [13] and foreign countries [14-16]. Interestingly, the approach was originally applied to prostate cancer incidence and mortality [10]. It helped determine when incidence started rising following the introduction of PSA test and estimate the amount of time diagnosis is advanced due to screening (lead time). On the other hand, a joinpoint model of cancer mortality was used to detect the possible benefits of PSA screening. Tests of comparison of joinpoint regression models were used by the same authors to compare female lung and breast cancer mortality rates between two registry areas and two states, respectively [12]. More recently, a similar methodology was applied to examine how socio-economic absolute and relative disparities in mammography use and associated changes in five breast cancer indicators varied over time [17]. A key difference was the use of rates that were smoothed using Bayesian hierarchical spatio-temporal methods, leading to more stable measures of disparities.

Most analyses of temporal trends have been aspatial and conducted at the National level or for a single cancer registry, with the implicit assumption that the trend parameters are constant across the study area. One exception is the study of temporal trends in breast cancer mortality by state and race from 1975 to 2004 [18]. Other authors also explored temporal trends in geographic disparities and classified 200 counties into priority groups based on changes in breast cancer incidence rates [19]. In a recent study on rates of low birth weight (LBW), joinpoint regression highlighted differences in temporal trends among the five geographical regions of Brazil [20]. Yet, no map was included in these studies, despite the benefit of mapping results of trend analysis.

Conducting a time series analysis through space raises several challenges, such as taking into account the instability of rates computed from smaller local populations, processing large amounts of results generated by multiple applications of joinpoint regression, or the inflated false discovery rate caused by multiple testing. This paper demonstrates how to apply the popular joinpoint regression approach at three different geographically nested levels: State, groups of metropolitan and non-metropolitan counties, and individual counties. Note that the analysis is conducted separately at each of these scales and does not involve a simultaneous evaluation of all three datasets.

The county-level analysis starts with a geostatistical pre-processing of the health data to filter their noise and ends with a post-processing that includes mapping of regression parameters and quantifying racial disparities in both space and time. In the first step, binomial kriging [21] allows one to capitalize on spatial autocorrelation and neighboring geographical units to filter the noise attached to health outcomes and to provide a measure of reliability (kriging variance) for the joinpoint regression. On the other hand, the combination of existing tests of comparison of time trends with multiple testing correction enables the application of joinpoint regression to the detection of geographical areas where time trends for the two races differ significantly. The disparity analysis is performed using a new statistic which measures the number of years where APC confidence intervals did not overlap. Summing up this statistic yearly over all geographical units provides an estimate of how the spatial extent of racial disparities changed with time. The approach is illustrated using times series of annual proportions of prostate cancer late-stage diagnosis available over the period 1981-2007 for each of the 67 counties in Florida.

## Methods

### Data

The data were downloaded from the Florida Cancer Data System website. They included the county-level incidence rates (used only to back-calculate population, see below) and number of cases of prostate cancer with associated stage at diagnosis that were recorded yearly from 1981 through 2007 for non-Hispanic white and black males. Proportions (rates) of late-stage diagnosis were computed over a 3-year moving window to reduce random fluctuations, yielding times series spanning 1982 through 2006. This computation was based only on cases 65 years and over to minimize the impact of disparities in age distribution across Florida and to attenuate the impact of variability in health coverage since all cases were covered by Medicare. The numbers of cases used in this study were: 206,993 white males and 19,442 black males.

Although a county-level analysis might seem rather crude and limits the interpretation of results because of potentially wide heterogeneity within a county, the present study represents a substantial improvement over most analyses of temporal trends which are usually aspatial and conducted at the National level or for a single cancer registry [22,23]. In addition, county-level analysis allowed the use of a fine temporal resolution (i.e. year) which would not be possible for finer spatial resolutions because of rate instability caused by the small number problem.

**Figure 1:**
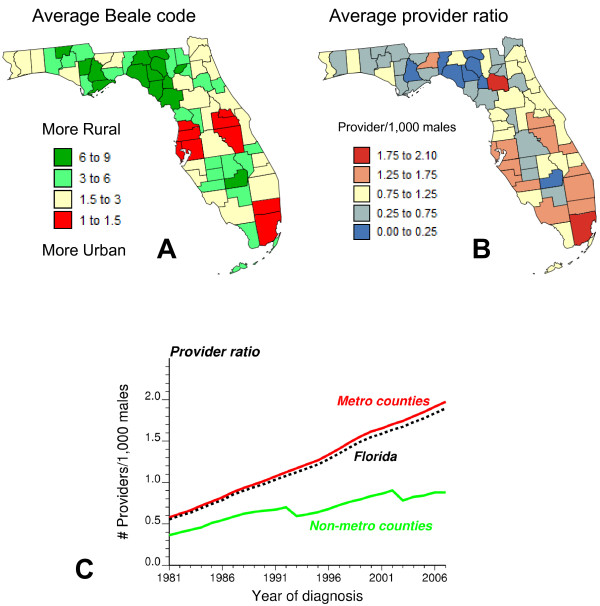
**Relationships between rural-urban continuum codes and provider ratios for Florida counties**. Rural-urban continuum codes, available for each county and three years (1983, 1993 and 2003), were linearly interpolated for each of the 27 years, and then averaged over the period 1981-2007 (A). A metropolitan county is characterized by a Beale code below 4. The provider ratio denotes the number of primary health providers and urologists per 1,000 habitants averaged at the county-level over 1981-2007 (B). The bottom plot shows how the provider ratio has increased with time for rural and non-rural counties (C).

Jemal *et al *[24] showed that non-metropolitan (non-metro) counties generally had higher death rates and incidence of late-stage disease and lower prevalence of prostate-specific antigen (PSA) screening test (53%) than metropolitan (metro) areas (58%). Following this study, results in this paper were interpreted on the basis of the US Department of Agriculture Rural-Urban Continuum Codes [25], which is most often referred to as the Beale codes, after its creator, Dr. Calvin Beale. This nine-part county codification distinguishes metro counties by the population size of their metro area, and non-metro counties by degree of urbanicity and adjacency to a metro area or areas (Table 1). This information was available for 1983, 1993 and 2003. For 1983 and 1993 codes 0 and 1 were combined to make these classifications comparable to the 2003's codification. These codes were linearly interpolated over the periods 1983-1993 and 1993-2003 to explore relationships between yearly health outcomes and urbanization. Figure 1A shows for each county the average urban code over the period 1981-2007. A metropolitan county is characterized by a Beale code of 3 or lower. The three metropolitan areas of Miami-Fort Lauderdale, Tampa and Orlando have the lowest Beale code. The more rural counties (highest Beale codes) are located in Northern Florida (Panhandle area) and in South Central Florida (Lake Okeechobee).

**Table 1:** Definition of 2003 Rural-Urban Continuum Codes (From http://www.ers.usda.gov/data/RuralUrbanContinuumCodes/).

Code	Description
*Metropolitan counties*	
**1**	Counties in metro areas of 1 million population or more
**2**	Counties in metro areas of 250,000 to 1 million population
**3**	Counties in metro areas of fewer than 250,000 population

*Non-metropolitan counties*	
**4**	Urban population of 20,000 or more, adjacent to a metro area
**5**	Urban population of 20,000 or more, not adjacent to a metro area
**6**	Urban population of 2,500 to 19,999, adjacent to a metro area
**7**	Urban population of 2,500 to 19,999, not adjacent to a metro area
**8**	Completely rural or less than 2,500 urban population, adjacent to a metro area
**9**	Completely rural or less than 2,500 urban population, not adjacent to a metro area

Beale codes can be used as a proxy for provider's accessibility. Health provider information was obtained from the Florida Department of Health Division of Medical Quality Assurance to calculate provider to population ratios. The data included the name and address of each provider (family and internal medicine, urology), the county where they practise, the original date where their license was activated, their license status and expiration date. If the expiration date was missing (case for all urologists), we assumed that the provider was still in practice. The number of primary health providers and urologists was computed for each county and each year over the time period 1981-2007. For the same years, the county-level population size for white and black males was back calculated from the raw incidence rates of prostate cancer. Whenever the rate was zero (i.e. no case diagnosed that year), the population was computed by linear interpolation between years where cases were diagnosed. The provider ratio was then computed for each county and year as the number of primary health providers and urologists active within that county during that year divided by the corresponding yearly county-level population. To visualize the spatial distribution of provider ratios over the State of Florida, these annual county-level ratios were averaged over all 27 years and the results, multiplied by 1,000, are mapped in Figure 1B. Counties with the highest provider ratio are Miami-Dade and Alachua which hosts the main campus of the University of Florida (Gainesville). Both maps (Figures 1A and 1B) display similar patterns. Figure 1C incorporates the time dimension and indicates that the number of providers per 1,000 habitants has steadily increased with time in metropolitan counties, widening the gap between rural and non-rural areas.

### Joinpoint regression

**Figure 2:**
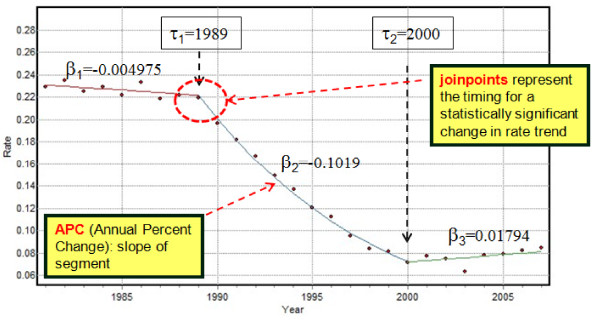
**Illustration of joinpoint regression model**. Annual proportions of prostate cancer late-stage cases were computed for white males 65 years and over that were diagnosed over the period 1981-2007 within Florida. The segmented regression model (solid line) includes two joinpoints (τ) that correspond to years of statistically significant changes in rate trend: 1989 and 2000. The parameters β are the slopes of the successive segments.

Let {*z*(*r*;*t*), *t *= 1,...,*T*} be the proportions or rates of late-stage diagnosis recorded for race *r *at *T *different time periods (e.g. years). Each observation *z*(*r*;*t*) is computed as the ratio *d*(*r*;*t*)/*n*(*r*;*t*), where *n*(*r*;*t*) is the total number of cases for race *r *at time *t*, and *d*(*r*;*t*) is the number of late stages. Joinpoint regression [10] models each time series as a sequence of linear segments (Figure 2). In its log-linear version, the segmented regression model is written as:

(1) Log(z(r;t))=μ(r;t)+ε(r;t)t=1,...,T

where *ε*(*r*;*t*) is the residual for the *t*-th time, and the regression mean *μ*(*r*;*t*) over the entire time interval [*a, b*] is modelled as a succession of (*K*(*r*)+1*) *linear segments (e.g. 3 segments in Figure 2): [*a*,τ_1_(*r*)] ... (τ_k_(*r*),τ_k+1_(*r*)] ... (τ_K_(*r*), *b*]. The parameter *τ*_k_(*r*) is the timing (joinpoint) for a statistically significant change in the slopes *β*_k_(*r*) and *β*_k+1_(*r*) of two successive segments.

#### Parameter estimation

The unknowns in the segmented regression model include the number *K*(*r*) and values *τ*_k_(*r*) of the joinpoints, the intercept *β*_0_(*r*), as well as the slope *β*_k_(*r*) of each segment. Their estimation is performed in two steps: 1) a grid search method [26] is conducted over the set of possible joinpoints, and 2) at each step of the search the regression parameters and their standard errors are estimated by weighted least-square regression using the following criterion:

(2) $$Q=\overset{T}{\underset{t=1}{\sum }}w\left(r;t\right){\left(\log\left(z\left(r;t\right)\right)-\mu \left(r;t\right)\right)}^{2}$$

The weighting scheme takes into account the fact that the variance of the residuals *ε*(*r*;*t*) typically varies with time (heteroscedasticity) as the number of cases changes. It is also an important issue when assessing racial disparities (see below) since at any given time there are usually much fewer minority cases. These weights are the reciprocal of the variance that can be computed, inside the NCI's Joinpoint regression program, if the dependent variable counts follow a Poisson distribution. The binomial distribution is more appropriate in the present study since late-stage diagnosis is not a rare event, and the weights were thus computed as *n*(*r*;*t*)/[*z*(*r*;*t*)×(1-*z*(*r*;*t*))]. In addition to being heteroscedastic, the random errors in the regression model could be autocorrelated. For example, the average correlation among residuals computed over all 134 time series (i.e. 67 Florida counties and 2 races) is 0.37 for Δt = 1 year, yet only -0.01 for Δt = 2 years. Uncorrelated error models were considered here since these are the only models available in the NCI software for testing the hypothesis of coincidence or parallelisms of different trend models used in the racial disparity analysis.

The number *K*(*r*) of joinpoints is estimated through an iterative procedure that tests whether models of increasing complexity (i.e. including more joinpoints) provide a significantly better goodness-of-fit than simpler models [27]. The tests of significance use a Monte Carlo Permutation procedure described in [10]. A maximum number of joinpoints is typically specified (i.e. *K*_max _= 3 here) to decrease the number of solutions and the computational time. To avoid that joinpoints get too close together or too close to either end of the time series, a minimum number of observations between joinpoints is also required and was set to 5 in the present application. This minimum number allows the computation of the standard error of the slope parameters, hence the calculation of their confidence intervals and the testing of whether these parameters are significantly different from zero.

#### Temporal trends

Trends in health outcomes recorded for race *r *over time interval [τ_k_(*r*), *τ*_k+1_(*r*)] can be quantified by the annual percent change (APC) that is calculated from the slope of the regression model over that time interval as:

(3) $$APC_{k+1}\left(r\right)=100\times \left(\text{exp}\left\{\beta_{k+1}\left(r\right)\right\}-1\right)$$

Like other regression parameters, confidence intervals can be computed for each APC and one can test whether an APC is significantly different from zero [10].

The trend over the entire time series [*a, b*] can be summarized by the average annual percent change (AAPC) that is the time-weighted average of the APC's from the joinpoint model: the weight of each APC is equal to the relative proportion of the time series [*a, b*] covered by the time interval [τ_k_(*r*), *τ_k+1_*(*r*)] . This measure is valid even if the joinpoint model indicates that there were changes in trends during those years [11]. Like for the APC, a (1-α) confidence interval can be computed and if it contains zero, then there is no evidence to reject the null hypothesis that the true AAPC is zero at the significance level of α.

#### Racial disparities

Disparities in temporal trends for two races *r *and *r' *can be detected by comparing the models fitted to their corresponding time series {*z*(*r*;*t*), *t *= 1, ...,*T*}and {*z*(*r'*;*t*), *t *= 1, ...,*T*}. Kim *et al*. [12] proposed a permutation procedure to compare two segmented line regression functions and to test two types of hypothesis: 1) the two regression models are identical, or 2) the two mean functions are parallel allowing different intercepts. In addition to these global tests, we conducted a finer comparison by computing for every time period *t *(i.e. year) the 95% confidence intervals of the APC for the two races and counting the number of times these two intervals did not overlap. This new racial disparity statistic can be expressed as:

(4) $$B_{rr^{\prime }}=\overset{T}{\underset{t=1}{\sum }}I\left(\text{CI}\left(r;t\right);\text{CI}\left(r^{\prime };t\right)\right)$$

where the indicator function I(.) = 1 if the following condition on the upper bounds (*U*) and lower bounds (*L*) of the two confidence intervals CI are met: *U*(*r*;*t*) <*L*(*r'*;*t*) or *L*(*r*;*t*) >*U*(*r'*;*t*). A large number indicates that rates of changes for the two races are consistently different over time. There is no statistical test associated with quantity *B_rr' _*which is mainly descriptive.

#### Geographical disparities

A spatial analysis of temporal trends can be conducted simply by applying the joinpoint regression to a set of *N *geographical units *v*_α_; for example the 67 counties within the State of Florida. For any unit *v*_α_, the regression model (1) is written as:

(5) $$Log\left(z\left(v_{\alpha };r;t\right)\right)=\mu \left(v_{\alpha };r;t\right)+\varepsilon \left(v_{\alpha };r;t\right)\;t=1,...,T$$

and all parameters are now race- and county-specific: *K*(*v*_α_;*r*), β_0_(*v*_α_;*r*), and{(β_k_(*v*_α_;*r*), τ_k_(*v*_α_;*r*)), *k *= 1,..., *K*(*v*_α_;*r*)}. Similarly, the estimation of temporal trend statistics (i.e. APC, AAPC) and the associated tests of hypothesis can be conducted for every geographical unit. Repeating the analysis across space allows exploring geographical disparities in temporal trends for each race separately but also investigating whether the magnitude of racial disparities changes across Florida. In particular, the racial disparity statistic (Equation 4) can now be computed for each geographical unit *v*_α _as:

(6) $$B_{rr^{\prime }}\left(v_{\alpha }\right)=\overset{T}{\underset{t=1}{\sum }}I\left(\text{CI}\left(v_{\alpha };r;t\right);\text{CI}\left(v_{\alpha };r^{\prime };t\right)\right)$$

Because the tests of hypothesis are conducted for each geographical unit, there is a great likelihood that some tests will turn out significant by chance alone (i.e. false positives), even if the null hypothesis of absence of racial disparity is true in all cases. Multiple testing corrections reduce the significance level applied to each test so that the overall false positive rate is kept to less than or equal to the user-specified significance level α. We used the false discovery rate (FDR) approach which was proven to be less restrictive and more powerful than other approaches, such as the simple Bonferroni correction [28].

As the size of the geographical units decreases, fewer cases are available for the computation of rates which become more unstable, in particular for minorities (small number problem). Thus, Goovaerts [Goovaerts P: Analysis of geographical disparities in temporal trends of health outcomes using space-time Joinpoint regression, submitted] proposed to replace each rate *z*(*v_α_*;*r*;*t*) and corresponding weight *ω*(*v_α_*;*r*;*t*) in the weighted least-square criterion (Equation 2) by the binomial kriging estimate $ẑ\left(v_{\alpha };r;t\right)$ and the inverse of the kriging variance $1∕\sigma_{BK}^{2}$ (*v*_α_;*r*;*t*). Binomial kriging was preferred over Poisson kriging [29] to acknowledge the fact that late-stage cancer diagnosis is not a rare event. The kriging estimate, which is a noise-filtered version of the original rate, is computed as a linear combination of the kernel rate *z*(*v_α_*;*r*;*t*) and the rates observed in (*n*-1) neighboring entities *v*_i _at that time *t*:

(7) $$ẑ\left(v_{\alpha };r;t\right)=\overset{n}{\underset{i=1}{\sum }}\lambda_{i}\left(r;t\right)z\left(v_{i};r;t\right)$$

The weights *λ_i _*(*r;t*) assigned to the *n *rates depend on the number of cases diagnosed within each county, the shape and size of the administrative units, as well as the pattern of spatial variability of the rates. These weights are solution of a system of linear equations, known as "binomial kriging" system; see [21,30] for more details.

#### Temporal disparities

Instead of looking at geographical or racial disparities in temporal trends, one could also explore temporal trends in the geographical extent of racial disparities. For example, the number of counties where the APC's confidence intervals for the two races did not overlap for any given time *t *is a measure of the geographical extent of racial disparities that existed at that time. This new geographical disparity statistic can be expressed as:

(8) $$D_{rr^{\prime }}\left(t\right)=\overset{N}{\underset{\alpha =1}{\sum }}I\left(\text{CI}\left(v_{\alpha };r;t\right);\text{CI}\left(v_{\alpha };r^{\prime };t\right)\right)$$

where the indicator function I(.) = 1 if the following condition on the upper bounds (*U*) and lower bounds (*L*) of the two confidence intervals CI are met: *U*(*v*_α_;*r*;*t*) <*L*(*v*_α_;*r'*;*t*) or *L*(*v*_α_;*r*;*t*) >*U*(*v*_α_;*r'*;*t*). A large number indicates that rates of changes for the two races were consistently different over a large part of Florida at that given point in time.

### Software

Joinpoint regression was conducted using the public-domain Joinpoint Regression Program 3.5.1 July 2011 [10] developed at the US National Cancer Institute, NCI (http://surveillance.cancer.gov/joinpoint/). Binomial kriging and multiple testing correction of *p*-values computed by Joinpoint Regression were performed using the commercial software SpaceStat 2.2 [31]. The three-dimensional display of county-level time series was created using SGeMS (Stanford Geostatistical Modelling Software [32]) 3D visualization panel and FORTRAN programs developed to format the data. All other computations, including the calculation of disparity statistics, were accomplished using FORTRAN programs developed by the first author.

## Results and discussion

### State-level analysis

**Figure 3:**
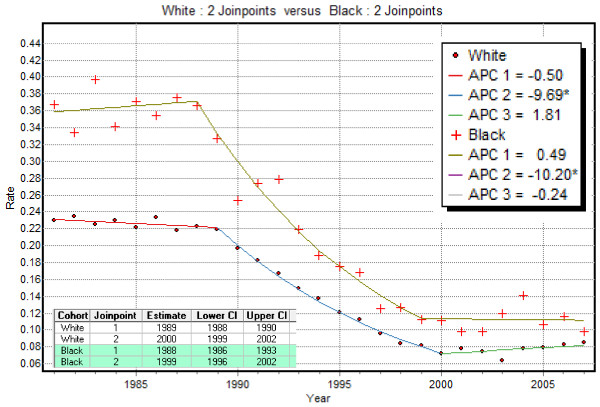
**Joinpoint regression model fitted to Florida time series of proportion of prostate cancer late-stage cases**. Annual proportions of prostate cancer late-stage cases were computed for non-Hispanic white and black males 65 years and over that were diagnosed over the period 1981-2007 within Florida. For both races, the segmented regression model (solid line) includes two joinpoints. Table 2 lists the estimate and 95% confidence intervals of the annual percent change (APC) for each segment, as well as the average annual percent change (AAPC) computed for the entire time period. Although the two curves are statistically non coincident, the hypothesis of parallelism of the two joinpoint regression models was not rejected at α = 0.05.

Figure 3 shows how the proportion of late-stage diagnosis changed yearly between 1981 and 2007 (*T *= 27) for non-Hispanic white and black males in Florida. Joinpoint regression models were fitted to each time series and the parameters are listed in Table 2. For all parameters, the confidence intervals are wider for black males because of the fewer cases: 19,442 black males versus 206,993 white males.

**Table 2:** Joinpoint regression analysis of state-level time series of proportion of prostate cancer late-stage diagnosis.

Parameter	White males			Black males		
	
	Estimate	CI_0.025 _	CI_0.975_	Estimate	CI_0.025 _	CI_0.975_
**Joinpoint**						
τ_1_	1989	1988	1990	1988	1986	1993
τ_2_	2000	1999	2002	1999	1996	2002
**APC**						
[1981, τ_1_]	-0.5	-1.6	0.6	0.5	-3.0	4.1
[τ_1_, τ_2_]	-9.7*	-10.4	-9.0	-10.2*	-12.2	-8.1
[τ_2_, 2007]	1.8	-0.2	3.8	-0.2	-4.3	3.9
**AAPC**						
1981-2007	-3.9*	-4.5	-3.3	-4.4*	-6.0	-2.7

Results for each ethnic group include the estimated value and 95% confidence interval for three parameters: joinpoint year, annual percent change (APC) for each of the three time segments, and average annual percent change (AAPC) computed over the entire time series. For the last two parameters, the symbol * denotes parameters significantly different from zero at α = 0.05.

Two joinpoints were fitted to each curve and the timing of significant changes for the two races was fairly similar. Both ethnic groups displayed a significant decline in the 90's when PSA screening test was introduced: Annual Percentage Change=-9.69% and -10.20%. The slopes of the two curves are non-significantly different since their confidence intervals overlap. During the two other time periods (late eighties and early 2000), time trends for white and black males were of opposite signs, albeit not significant. Over the entire time period, the average annual rate of change was slighter greater for black (AAPC = -4.4%) than for white (AAPC = -3.9%) although once again the difference was not statistically significant. The racial disparity statistic *B_rr' _*(Equation 4) equalled 2 yrs since the APC confidence intervals did not overlap in 1999 and 2000.

### Urban versus rural areas

The impact of urbanization on temporal trends was explored by grouping, every year, the sixty seven counties based on whether their interpolated Beale index exceeded 3 (non-metropolitan or rural group) or was below 4 (metropolitan or urban group). For both races, the percentage of cases in rural counties decreased with time but these cases tend to be diagnosed at later stages than in more urbanized counties, which confirms results by Jemal *et al*. [24].

**Figure 4:**
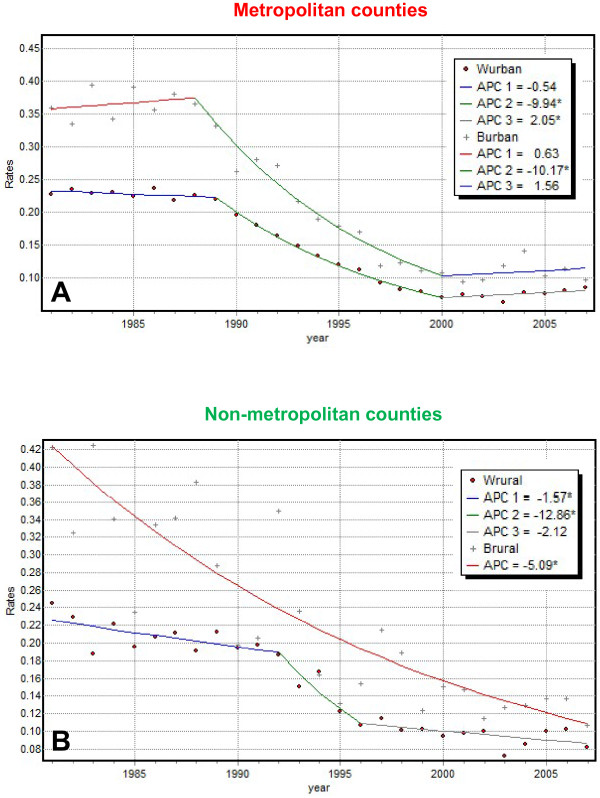
**Joinpoint regression models fitted to proportions of prostate cancer cases diagnosed late in metropolitan and non-metropolitan counties**. Annual proportions of prostate cancer late-stage cases were computed for non-Hispanic white and black males 65 years and over that were diagnosed over the period 1981-2007 in metropolitan and non-metropolitan counties. Table 3 lists the estimate and 95% confidence intervals of the annual percent change (APC) for each segment, as well as the average annual percent change (AAPC) computed for the entire time period. Although the curves for white and black males are statistically non coincident, the hypothesis of parallelism of the two joinpoint regression models was not rejected at α = 0.05 in both metro and non-metro counties.

Joinpoint regression analysis was conducted for both races and both groups of counties. Parameters of the regression models displayed in Figure 4 are listed in Table 3. Because about 90% of cases were on average diagnosed in metropolitan counties, the models fitted to the metro time series looked (Figure 4A) very similar to the models fitted for Florida (Figure 3). A major difference was that both races now displayed an increase in proportion of late-stage diagnosis for the last time period, and this trend was significant for white males. This result went undetected during the State-level analysis. The hypothesis of parallelism of the two regression models was not rejected (*p*-value = 0.461) and the racial disparity statistic equalled 1 since the APC confidence intervals did not overlap in 1989.

**Table 3:** Joinpoint regression analysis of proportions of prostate cancer cases diagnosed late in metropolitan and non-metropolitan counties.

Parameter	White males			Black males		
	
	Estimate	CI_0.025 _	CI_0.975_	Estimate	CI_0.025 _	CI_0.975_
	*Metropolitan counties*					
**Joinpoint**						
τ_1_	1989	1988	1990	1988	1985	1992
τ_2_	2000	1999	2002	2000	1999	2002
**APC**						
[1981, τ_1_]	-0.5	-1.6	0.6	0.6	-2.9	4.3
[τ_1_, τ_2_]	-9.9*	-10.7	-9.2	-10.2*	-12.0	-8.3
[τ_2_, 2007]	2.1*	0.1	4.1	1.6	-3.6	6.9
**AAPC**						
1981-2007	-4.0*	-4.6	-3.3	-4.3*	-6.0	-2.5

	*Non-metropolitan counties*					
**Joinpoint**						
τ_1_	1992	1989	1994	-	-	-
τ_2_	1996	1994	2003	-	-	-
**APC**						
[1981, τ_1_]	-1.6*	-2.9	-0.2	-	-	-
[τ_1_, τ_2_]	-12.9*	-23.4	-0.8	-	-	-
[τ_2_, 2007]	-2.1	-4.3	0.1	-	-	-
**AAPC**						
1981-2007	-3.6*	-5.7	-1.5	-5.1*	-6.2	-3.9

Results for each ethnic group include the estimated value and 95% confidence interval for three parameters: joinpoint year, annual percent change (APC) for each of the linear time segments, and average annual percent change (AAPC) computed over the entire time series. For the last two parameters, the symbol * denotes parameters significantly different from zero at α = 0.05.

Since fewer cases were diagnosed in non-metro counties, their time series were more irregular and confidence intervals for a few parameters widened greatly. No joinpoint was estimated for black males whose time series was fitted using a single exponential curve. For white males, all the linear segments had negative slopes which were significantly different from zero over the first two time periods. This continuous decline in non-metro counties was a significant departure from the recent increase observed in metropolitan counties. This was confirmed by the rejection at α = 0.05 of the hypothesis of parallelism of joinpoint regression models fitted to rates for white males in metro and non-metro counties. This hypothesis was not rejected for any comparison involving black males because of the uncertainty caused by smaller population sizes. The racial disparity statistic (Equation 4) was much larger in rural counties (*B_rr' _*= 12 yrs) than in urban counties (*B_rr' _*= 1 yr).

### County-level analysis

The above analysis of temporal trends in metro and non-metro areas revealed the existence of geographical and racial disparities that could not be detected at the State level. An even finer spatial analysis could be performed by examining the county-level time series of proportions of late-stage diagnosis. Such an analysis needed however to be conducted after smoothing using binomial kriging because of the larger rate instability observed when moving to smaller geographical units. Another reason for the application of smoothing techniques was the existence of missing values (i.e. years where no case was diagnosed) that needed to be replaced by rate estimates in order to run NCI joinpoint regression program. Missing values were only observed for black males and represented 6.27% of all rate-years. Binomial kriging was conducted using as neighbours the counties that share a common border or vertex with the county being smoothed (1^st ^order Queen's adjacency). A population-weighted variogram of percentage of late-stage diagnosis was computed every year for both races. The fitted variogram model had on average a longer range of autocorrelation for white males (110 km) than for black males (85.5 km).

#### Visualization of geographical and temporal disparities

**Figure 5:**
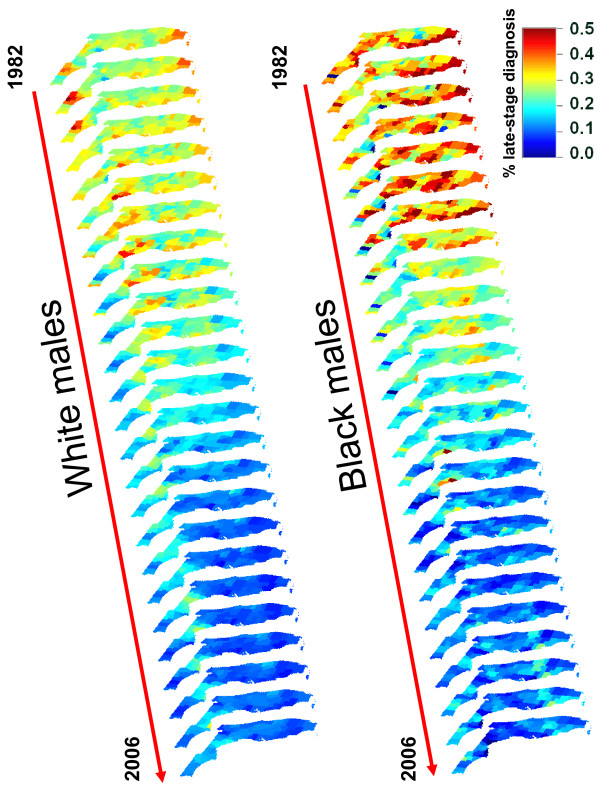
**Three-dimensional representations of 25 maps of county-level proportions of late-stage prostate cancer for white and black males in Florida**. The fill color in each county represents the proportion of late-stage prostate cancer aggregated within 3 year windows and noise-filtered using binomial kriging at the county level for two ethnic groups (cases 65 yr and older). The same color scale is used for all the maps that were aligned, according to the year of records 1982 through 2006, along a time axis rotated to minimize slide overlaps.

The three-dimensional representation of Figure 5 allowed a joint visualization of geographical, racial, and temporal disparities. The use of the same color scale for both ethnic groups emphasized the largest proportion of late-stage diagnosis for black males in particular in the eighties. For white males, this display highlighted the Florida Panhandle where proportions were consistently high in the Big Bend region whereas they were much lower in the adjacent Tallahassee area. The trend was intermediate in Central and South Florida where late-stage diagnosis has been declining since the mid nineties. In South Florida, percentages appeared however to remain high for a longer time on the West coast relative to the East Coast. A similar East-West trend was observed for black males in South Florida, but the Big Bend region does not stand out as much as for white males.

**Figure 6:**
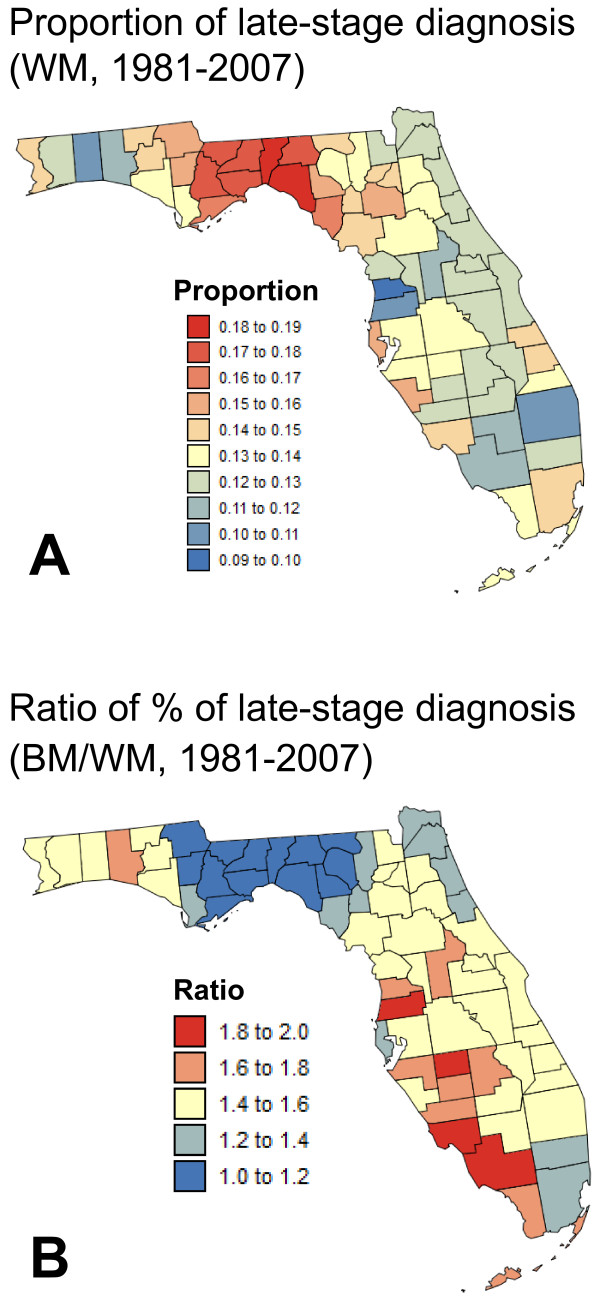
**Time-average proportions of prostate cancer late-stage diagnosis for white and black males**. The yearly kriging estimates were averaged over the period 1982-2006 and weighted according to the inverse of the binomial kriging standard deviations to assign more importance to more reliable estimates. For black males (BM), results are expressed as the ratio of average proportions for black versus white males (WM) to facilitate the visualization of racial disparities.

To facilitate the visualization of geographical disparities, the proportion of late-stage diagnosis was averaged over the entire time period and mapped in Figure 6. To account for the small number problem, the averaging was conducted over the noise-filtered rates and each rate was weighted by the inverse of the binomial kriging standard deviation. Figure 6A confirmed the interpretation of the three-dimensional graph of Figure 5: average proportions of late-stage diagnosis for white males were large in the Florida Panhandle, in particular in the Big Bend region. In that region, proportions were of similar magnitude for black males (ratio close to 1, Figure 6B) whereas racial disparities were the largest along the West Coast of South Florida where rates of late-stage diagnosis were 60 to 100% larger for black males compared to white males.

#### Analysis of heavily populated counties

**Figure 7:**
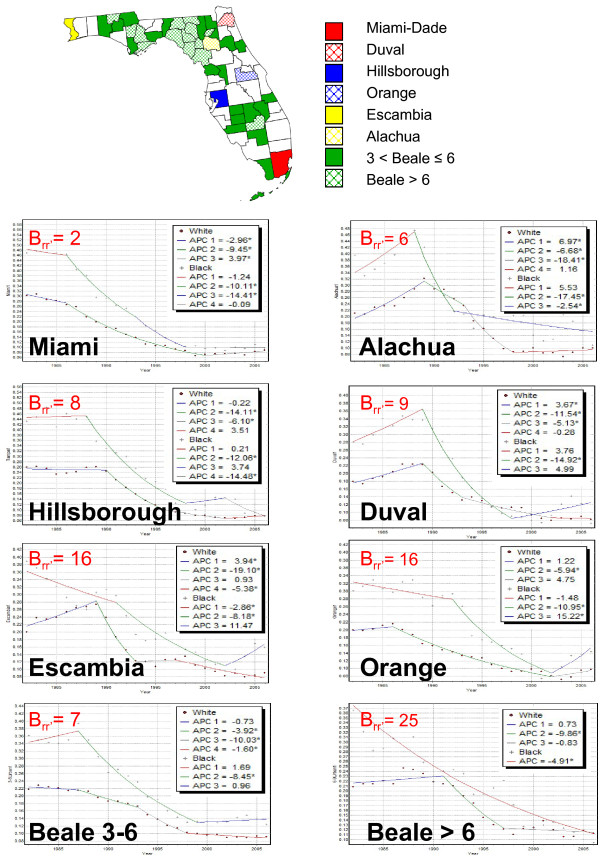
**Joinpoint regression models fitted to proportions of prostate cancer cases diagnosed late in several Florida counties**. Joinpoint regression was conducted on the time series of noise-filtered proportions of late-stage diagnosis recorded in six heavily populated counties and the two groups of rural counties identified in Figure 1A. Within each county or group of counties, the hypothesis of parallelism of models for white and black males was rejected at α = 0.05 and the disparity statistic *B_rr' _*was computed.

Geographical disparities were first investigated by looking at the time series of proportions of late-stage diagnosis for both white and black males in six heavily populated metro counties and the two groups of rural counties identified in Figure 1A. The location of these counties is displayed at the top of Figure 7. For both races, the largest percentage of late-stage diagnosis in the eighties occurred in Miami-Dade, followed by Hillsborough (Tampa), whereas the lowest percentages were observed in Duval County (Jacksonville), followed by Orange County (Orlando). Surprisingly, Miami-Dade and Hillsborough are the counties with some of the highest provider ratio, which does not support the hypothesis that screening access would impact significantly late-stage diagnosis. In the nineties, differences between counties started decreasing for the health outcomes but widening for the provider ratio (results not shown), another indication that geographical disparities cannot be explained by differences in screening access. It is noteworthy that as the proportion of late-stage diagnosis decreased with time, the largest percentages of late-stage cases shifted to rural counties relative to urban counties.

Racial disparities within these same geographical units were investigated by conducting a joinpoint regression analysis and testing the hypothesis of parallelism of regression models for both races. Figure 7 showcased some of the geographical and racial disparities observed over Florida. Although all geographical units and races experienced a significant decline in percentage of late-stage diagnosis during the nineties, joinpoint regression models differed greatly. For some counties, like Alachua or Duval County, this decline followed a significant increase in the eighties while for other counties the rates remained stable or slightly decreased during that time period. Similarly, a wide spectrum of temporal trends was observed for the most recent years; for example for white males a significant decline in Escambia County contrasted with a significant increase in Miami-Dade County. The hypothesis of parallelism was rejected at α = 0.05 for all six counties and two groups of rural counties. The use of the disparity statistic *B_rr' _*(Equation 6) allowed discriminating the different geographical units based on the frequency of racial disparities in annual percent changes (APC). Figure 7 indicates that disparities were the smallest for Miami-Dade County (*B_rr' _*= 2 yrs) where both curves had similar joinpoints and slopes of similar sign. Racial disparities in temporal trends are much larger for Escambia and Orange Counties (*B_rr' _*= 16 yrs), and reached a maximum for the group of very rural counties (*B_rr' _*= 25 yrs). In the later case no joinpoint was estimated for black males whose time series was fitted using a single exponential curve with significant APC. For white males, the decline was only significant between 1991 and 1997. Note that since the racial disparities in APC mostly (19 years out of 25 years) took the form of larger declines for black males compared to white males, the racial disparities in percentage of late-stage diagnosis at the end of the time period are actually one of the smallest among all geographical units.

#### Analysis of all Florida counties

**Figure 8:**
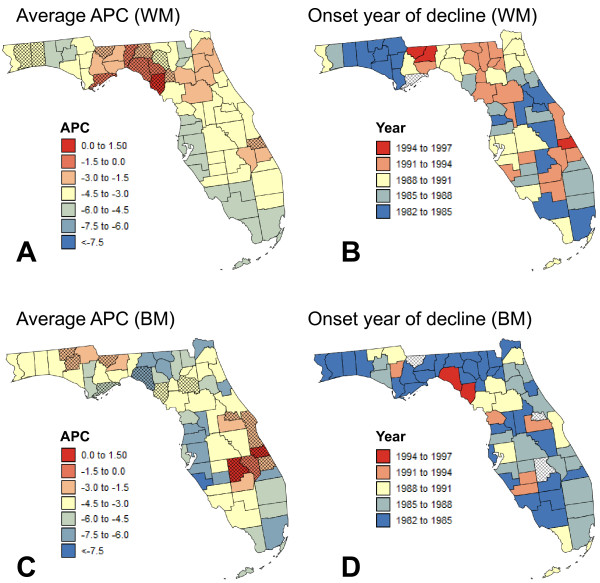
**Onset years for first significant declines in county-level proportions of late-stage diagnosis and Average APC over 1981-2007**. For each ethnic group and county in Florida, two statistics were computed from the joinpoint regression models fitted to the time series of noise-filtered proportions of prostate cancer late-stage diagnosis: onset year for the first significant decline in the proportion of late-stage diagnosis (i.e. APC negative and significantly different from zero), and average annual percent change (AAPC). Shaded counties denote counties without significant decline or AAPC not significantly different from zero.

The same analysis was conducted for each of the 67 Florida counties in order to explore the geographical distribution of racial disparities in more details. The geographical variability of temporal trends was summarized using two statistics mapped in Figure 8: the average annual percent change (AAPC) and the joinpoint corresponding to the first significant decline in proportion of late-stage diagnosis (i.e. negative APC significantly different from zero). The annual rate of decrease in prostate cancer late-stage diagnosis and the onset years varied greatly across Florida and among racial groups. The use of the same colour scale for the two racial groups in Figure 8 made clear that compared to white males, black males experienced a larger average annual percent decline in late-stage diagnosis over the period 1981-2007 and that this decline started earlier.

For white males, most counties with non-significant AAPC were located in the Florida Panhandle, which explained the largest proportion of late-stage diagnosis observed on average over the entire time period. For black males, counties with non-significant AAPC clustered in southeast Florida. Both races experienced the largest overall decline in percentage of late-stage diagnosis in Miami-Dade County and Central-west Florida (Tampa Bay); this decline started earlier for Miami-Dade County, in particular for white males (Figure 8B-D). Among the three metropolitan areas with the lowest Beale codes (Figure 1A), Orlando area (Orange county) showed the least progress in lowering the frequency of late-stage diagnosis, yet the initial proportion of late-stage cases was smaller in that county. Surprisingly, Alachua County (University of Florida) that has the second largest provider ratio in the State (Figure 1B) has a very low AAPC, which is not significantly different from zero for black males. The Florida Panhandle encompassed large differences in onset years for white males: significant declines in frequency of late-stage diagnosis started much later in the Big Bend region than in the ten counties west of it.

#### Racial disparities

**Figure 9:**
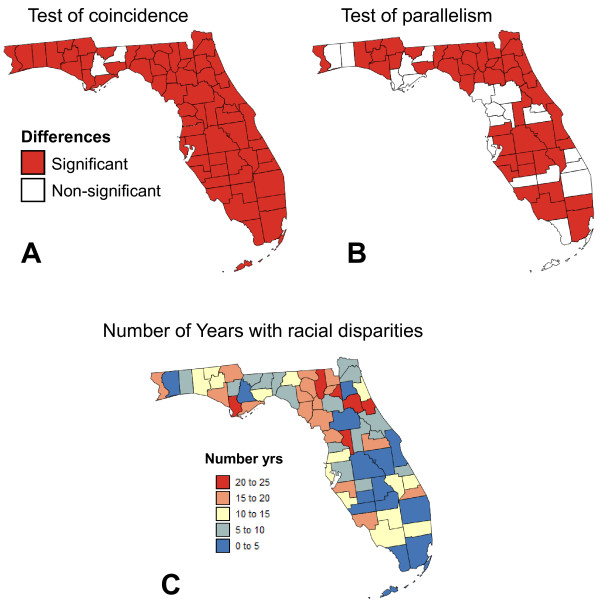
**Spatial and temporal distributions of significant racial disparities for prostate cancer late-stage diagnosis**. Coincidence and parallelism of county-level time series of proportion of prostate cancer cases diagnosed late for white and black males were tested using a significance level α = 0.01. Multiple testing corrections were performed using the False Discovery Rate (FDR) approach. White polygons depict non-significant (NS) differences. Map (C) displays the number of years where the 95% confidence intervals of APC for white and black males did not overlap (Equation 6).

Coincidence and parallelism of county-level time series of proportion of prostate cancer cases diagnosed late for white and black males were tested using a significance level α = 0.01. Multiple testing corrections were performed using the False Discovery Rate (FDR) approach. Figure 9A indicated that time series for white and black males differed significantly in 65 counties out of 67. Hypothesis of parallelism was rejected in fewer counties: 20 out of 67 (Figure 9B). Interestingly, counties with parallel time series tended to cluster, such as in the Big Bend region or North of Tampa Bay. A third measure of discrepancies between Whites and Blacks' time series was the disparity statistic (Equation 6) that measured the number of years where the 95% confidence intervals of APC for the two races did not overlap. Counties where the two races frequently displayed significant difference in APC values were mainly rural and located between the Big Bend region and Alachua County that hosts the campus of the University of Florida (Figure 9C). Metropolitan counties (Beale index ≤ 3) experienced significant disparities for 8.4 yrs on average, whereas for non-metro counties the B_rr' _statistic equalled 12.8 yrs. This result confirms the conclusions drawn from Figure 4 where time series for non metro counties had much different shapes (B_rr' _= 12 yrs) than metro counties (B_rr' _= 1 yr).

### Sensitivity analysis

**Figure 10:**
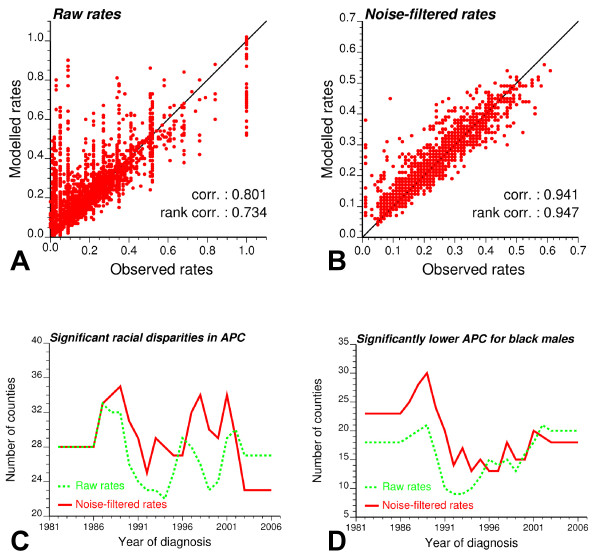
**Impact of using noise-filtered rates versus raw rates on the results of joinpoint regression**. The scatterplots of observed versus modelled rates were created for joinpoint linear regression models fitted to raw rates (A) or rates that were processed using binomial kriging (B). Bottom plots illustrate the impact of noise filtering on the temporal trend of two measures of racial disparities: (C) the number of counties where the 95% confidence intervals of APC for white and black males did not overlap (Equation 8), and (D) the number of counties where APC for Blacks is significantly smaller than for Whites.

One novelty of the county-level analysis was the use of binomial kriging to filter out noise before applying joinpoint regression. To explore the impact of such filtering on the results, joinpoint regression was also performed on the time series of raw rates. Missing values for black males (105 values = 6.27% of all rate-years) were first replaced by the following rates: 1) State-wide rates for 1982's missing values, and 2) rate observed the prior year for all values that were missing in 1983 and after. The scatterplots in Figures 10A-B illustrated the closer fits of the joinpoint regression models inferred from noise-filtered rates versus raw rates: the correlation between observed and modelled values increased from 0.80 to 0.94 after application of binomial kriging. Raw rates are much more variable (i.e. larger variance) and their use in joinpoint regression resulted in larger bias: residual mean is -0.029 compared to -0.005 for noise-filtered rates.

The second comparison criteria were the two summary statistics mapped in Figure 8: the average annual percent change (AAPC) and the joinpoint corresponding to the first significant decline in proportion of late-stage diagnosis (i.e. onset years). For both parameters, the use of raw rates yields a wider range of values because of the lack of reliability of time series recorded for sparsely populated counties. When looking at counties with at least 5 cases per year on average, the AAPC computed from joinpoint regression of raw rates and noise-filtered rates are relatively similar: the linear correlation coefficient is 0.62 for white males (54 counties) and 0.69 for black males (23 counties). Smaller correlation coefficients were observed for onset years, in particular for black males: 0.45 versus 0.68 for white males. Although the underlying temporal trends are unknown, simulation studies [21,29] have demonstrated the benefit of noise-filtering by kriging to estimate the "true" cancer risks relatively to the use of raw estimates. We can thus hypothesize that the greater prediction accuracy of kriging translates into a more accurate modelling of temporal trends.

The impact of noise filtering on the detection of racial disparities by the new disparity statistic *D_rr' _*(Equation 8) was also explored. This statistic measures how the geographical extent of significant racial disparities changed yearly over the period 1981-2007. Figure 10C showed that the number of counties with significant racial disparities peaked around 1990, because the decline started earlier for black males. Both approaches lead to similar conclusions although the 1990's peak was more apparent when analyzing raw rates. The time series in Figure 10D revealed that the percentage of counties with more favorable changes for black males (i.e. significantly smaller APC) sharply dropped in the early nineties when PSA screening was introduced. In this case, the use of noise filtered rates enhanced the 1990's peak. For both statistics, using raw rates led on average to the detection of fewer significant disparities because of the uncertainty attached to these rates.

### The MAUP effect

**Figure 11:**
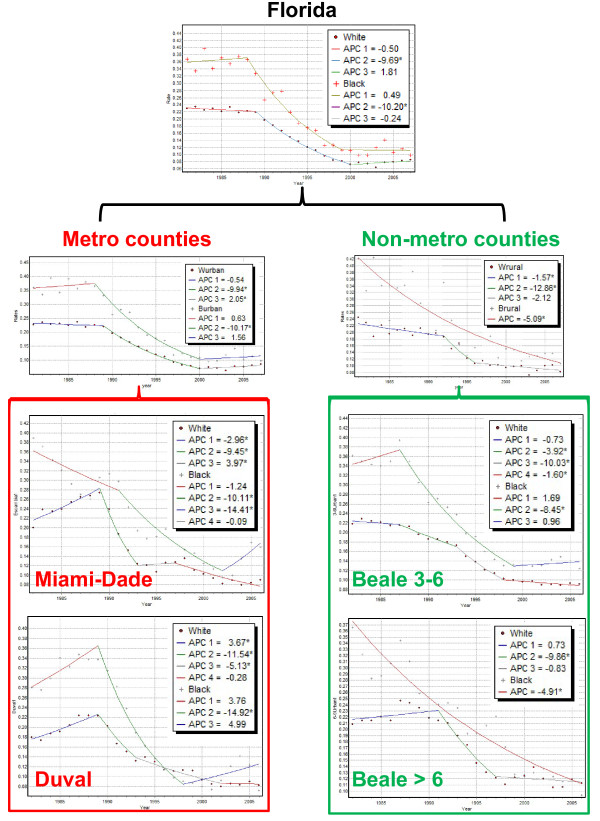
**Illustration of multi-scale joinpoint regression analysis**. This figure includes results of joinpoint regression analysis displayed in Figures 3, 4 and 7. The regression analysis was conducted for geographical units defined at three nested levels of spatial resolution: 1) State of Florida, 2) two groups of metropolitan and non-metropolitan counties, and 3) two heavily populated metro counties plus the two groups of rural counties identified in Figure 1A.

An originality of the present study was the application of joinpoint regression at three geographically nested levels: State of Florida, groups of metropolitan and non-metropolitan counties, and individual counties. The main results observed at each scale were summarized in Figure 11 and Table 4 that illustrate the modifiable areal unit problem (MAUP) whereby different geographic scales lead to inconsistent results for health outcomes [33,34]. As the size of geographical units decreases, temporal trends in late-stage diagnosis for white and black males become increasingly different, in particular in rural areas, which denote the existence of both a scale effect and a zoning effect. The magnitude of racial disparities, as measured by the new disparity statistic B_rr' _(Equation 6), greatly increased when the analysis was conducted on cases diagnosed in non-metropolitan Florida (Figure 11): the number of years with non-overlapping confidence intervals for APC jumped from 2 to 12. Dividing this geographical unit based on whether the county-level Beale index exceeds 6 or not enhanced these disparities even more, with the disparity statistic reaching a maximum of 25 years for the most rural counties (Table 4). On the other hand, this stratification of the State based on the Beale index revealed a greater similarity between races in urban areas (B_rr' _= 1); an interesting result was the recent increase in the frequency of late-stage diagnosis shared by both races. The county-level analysis however showed a great heterogeneity among cities. For example, racial disparities in Miami-Dade (B_rr' _= 2) were much smaller than in Orange county (B_rr' _= 16).

**Table 4:** Impact of geographical scale on parameters of the joinpoint regression models and detection of racial disparities.

Geographical	AAPC		Onset year		Racial disparities	
	
Units	WM	BM	WM	BM	H_o _: Parallel	B_rr' _statistic
**State**						
Florida	-3.9	-4.4	1989	1988	NR	2
						
**Metro vs Nonmetro**						
Urban counties	-4.0	-4.3	1989	1988	NR	1
Rural counties	-3.6	-5.1	1982	1982	NR	12
						
**Counties**						
Miami-Dade	-5.2	-6.4	1982	1986	R	2
Duval	-3.0	-3.3	1989	1989	R	9
Hillsborough	-4.8	-7.0	1990	1988	R	6
Orange	-3.1	-3.0	1986	1992	R	16
Escambia	-4.2	-3.1	1989	1982	R	16
Alachua	-3.0	-3.3	1982	1988	R	6
Beale 3-6	-3.8	-3.7	1987	1987	R	7
Beale > 6	-2.6	-4.9	1991	1982	R	25

Results for white males (WM) and black males (BM) include: 1) the average annual percent change (AAPC) computed over the entire time series, 2) the onset year for the first significant decline in the proportion of late-stage diagnosis (i.e. APC negative and significantly different from zero), 3) the results of the test of parallelism using a significance level α = 0.05 (R = reject, NR = non reject), and 4) the value of the *B_rr' _*statistic that measures the number of years where the 95% confidence intervals of APC for white and black males did not overlap (Equation 6).

In their study on breast and prostate cancer survival in Michigan over the period 1985-2002, Meliker *et al*. [35] used changes in the magnitude of absolute and relative disparity statistics across geographic scales to evaluate the relative importance of innate and societal-level factors in explaining racial disparities. Unlike in the present study, racial disparities diminished and virtually disappeared in smaller geographic units (state House districts and urban neighborhoods) where the population at risk is more uniform with regard to modifiable risk factors, such as socio-economic status and proximity to screening facilities. The authors concluded that modifiable factors were responsible for apparent racial disparities observed at larger scales. The opposite trend was observed in another study [36] on the detection of disparities in breast cancer mortality among three ethnic groups in Texas (period 1995-2005). The frequency of racial disparities increased when moving down from the county level to the ZIP code and census tract levels. According to the authors, this may indicate that contextual and environmental risk factors exert different roles on health at different aggregation levels. Another culprit was the attenuation of health difference within larger geographic regions where the impact of population concentration and racial residential segregation of minorities into small and specific areas is diluted. A similar reasoning could apply here where the mixing of patients diagnosed in rural and urban areas tend to blur differences between races at the State level. However the analysis of time series distinguishes the present study from other analyses of the impact of spatial scale on racial disparities that were static and conducted for a single time period.

One solution to the MAUP effect is the use of disaggregated data [37]. Census tract level rates are the most spatially detailed data available for the present application. Thus, the creation of a continuous map of proportion of late-stage diagnosis would require a disaggregation of these data, for example using area-to-point binomial kriging [38]. For Oppenshaw and Rao [39], the answer to the MAUP effect resides in the creation of zones of approximately equal population size, or tailored to standardize the results of specific analyses. This has led to the development of automated zone matching (AZM) methodology [40] for automated zone design. For example, in a recent study on low birth weight and infant mortality in Michigan [41] AZM was used to create aggregates of ZIP codes that meet a series of constraints, such as a minimum number of cases per unit, spatial compactness and maximum intra-area correlation to ensure homogeneity in terms of race and educational level. A similar approach was used here since counties were aggregated based on their Rural-Urban Continuum Codes which proved to be an important factor in explaining the magnitude of racial disparities.

The county-level joinpoint regression analysis revealed large differences among counties within the same zone, for example urban counties (Figure 7 and Table 4), which reflects the spatial non-stationarity of the parameters of the time trend models. Geographically-weighted regression [37] was developed to account for the spatial variability of regression parameters. In this approach the regression analysis is conducted within local windows where each observation is weighted according to its proximity to the centre of the window. Such locally varying models may not be influenced by MAUP issues to the same extent as the global regression models that ignore the spatial location of the data [37]. Future research should explore the generalization of this concept to joinpoint regression models.

## Conclusions

Too often racial disparities in health outcomes are evaluated simply by computing the difference or ratio between crude rates, ignoring the lack of reliability of rates recorded for small minority populations, as well as the spatial and temporal dimensions of the data.

This paper went beyond earlier methodological work on quantifying racial disparities [35,36,42] by incorporating the temporal dimension through the comparison of time series instead of rates aggregated over a given time period. This comparison relied on the innovative application of joinpoint regression to rates that were stabilized using binomial kriging. A second innovation was the introduction of a new disparity statistic to supplement the tests of parallelism and coincidence available in joinpoint regression for the comparison of time series. A major advantage of this disparity statistic, which measures the number of years where APC confidence intervals did not overlap, is that it can be summed up yearly over all geographical units, providing an estimate of how the spatial extent of racial disparities changed with time. It is noteworthy that unlike previous disparity measures this statistic quantifies disparities in the pace of changes instead of the values of the health outcomes themselves. Within the context of cancer control and surveillance, this statistic facilitates the quantification of how health outcomes for different racial groups changed following strategies to improve cancer prevention and early detection, which should help better understand the causes underlying observed racial disparities in cancer incidence, mortality and morbidity.

A major challenge when working in both the spatial and temporal domains is the issue of scale or resolution. An accurate determination of when the slopes of the linear models fitted to time series undergo statistically significant changes (i.e. number and location of joinpoints) requires data that are reliable and with a good temporal resolution. Joinpoint regression has been traditionally applied to yearly time series at the State or Federal level. Keeping the same temporal resolution while zooming into the county level for example enhanced the small number problem; in particular as the focus is here on minority populations that represent on average only 10% of cases. The issue of rate instability was tackled by using both the rate estimates and standard errors provided by binomial kriging as input to joinpoint regression. A sensitivity analysis showed that kriging-based noise-filtering improved the fit by the joinpoint regression models (i.e. lower residual variability) compared to the modelling of raw rates. Another benefit of using noise-filtered rates was the clearer detection of the drop in the percentage of counties with more favorable changes for black males (i.e. significantly smaller APC) that occurred in the early nineties when PSA screening was introduced.

Another issue associated with the spatialization of joinpoint regression results is the repetition of tests of hypothesis that need to be conducted for each geographical unit, increasing the risk of false positives. Multiple testing correction was here applied using the traditional implementation of the false discovery rate (FDR) approach which is based on the underlying assumption of independence of tests. This assumption might not be appropriate for adjacent geographical units whose kriged estimates are based on common neighbors. Several techniques were proposed recently to account for highly correlated test statistics in the FDR approach [43,44]. These approaches might however be too conservative since tests for geographical units that are further apart are independent and only adjacent counties were used in binomial kriging. In addition, some authors [28] hypothesized that the spatial dependence could be controlled by the traditional FDR correction given its statistical properties. More research is needed on this issue of multiple testing correction [45].

The case-study illustrated very well how the proportion of late-stage diagnosis for a common disease, such a prostate cancer, can change dramatically over time (i.e. 50% decline over 20 years) and display striking geographical and racial disparities within a single State. Thus, a comprehensive picture of the burden of cancer and the impact of various interventions can only be achieved through the simultaneous incorporation of the spatial and temporal dimensions in the visualization and analysis of health outcomes and putative covariates. State-level percentage of late-stage diagnosis decreased 50% since 1981; a decline that started slightly earlier for black males which had historically high rates of late-stage diagnosis. This decline accelerated in the 90's when PSA screening was introduced and stopped abruptly in the early 2000. Analysis at the metropolitan and non-metropolitan levels revealed a recent urban increase in the frequency of late-stage diagnosis for both races, and this trend was significant for white males. This result has important public health implications since it might cause an increase in prostate cancer mortality in the future. Non-metropolitan counties, despite displaying a steady decline in percentage of late-stage diagnosis, are still behind urban areas that generally have higher providers to cases ratio. Larger differences in temporal trends for Whites and Blacks were also observed in these rural counties, as measured by the new disparity statistic. In particular, the group of counties with a Beale index above 6 (i.e. most rural counties) received the maximum value for the racial disparity statistic, indicating that the yearly rate of change was significantly different between races for each of the 25 years.

The annual rate of decrease in prostate cancer late-stage diagnosis and the onset years for significant declines varied greatly among counties and racial groups. This spatial heterogeneity reflects the non-stationarity of the parameters of the time trend model even within the metropolitan and non-metropolitan strata and might warrant the development of a geographically-weighted version of joinpoint regression. Most counties with non-significant average annual percent change (AAPC) were located in the Florida Panhandle for white males, whereas they clustered in south-eastern Florida for black males. The new disparity statistic indicated that the spatial extent of racial disparities reached a peak in 1990 because of an early decline in frequency of late-stage diagnosis observed for black males. This result suggests the existence of racial disparities in the application or availability of PSA testing, in particular as the new screening procedure was introduced.

The present study was mainly methodological and the interpretation of the results suffers from limitations typically associated with ecological studies. As discussed before, the modelling of temporal trends requires some level of spatial aggregation in order to capture enough cases for a reliable estimation of percentages of late-stage diagnosis on an annual basis, which is the main culprit for the MAUP effect. In addition, the use of cases 65 year old and older allowed controlling for one source of individual-level heterogeneity because the entire study population had Medicare coverage. Individual-level data available for the same period are being analyzed to explore the impact of individual characteristics, area-level census measures of education, income, and environmental exposure on prostate cancer mortality, incidence and stage at diagnosis. These data will help conduct joinpoint regression at the sub-county level for heavily populated areas where enough cases are available for geographically detailed analysis and modelling.

## Competing interests

The first author is affiliated with BioMedware a research company that also develops software for the exploratory spatial and temporal analysis of health and environmental data. With funding from the National Cancer Institute, the authors developed the SpaceStat software that includes binomial kriging.

## Authors' contributions

PG carried out all the analysis and drafted the manuscript. HX participated in the design of the study. All authors read and approved the final manuscript.
